# Appraisal of ChatGPT's responses to common patient questions regarding acromioclavicular joint dislocations

**DOI:** 10.1016/j.xrrt.2025.06.020

**Published:** 2025-08-05

**Authors:** Eduard Van Eecke, Wouter Schroven, Maxim Vanderstappen, Simran Grewal, Michel P.J. van den Bekerom

**Affiliations:** a Department of Orthopaedic Surgery, AZ Delta, Roeselare, Belgium; b Department of Orthopaedic Surgery, UZ Leuven, Leuven, Belgium; c Shoulder and Elbow Unit, Department of Orthopaedic Surgery, OLVG, Amsterdam, The Netherlands; d Department of Orthopaedic Surgery, AZ Monica, Antwerp, Belgium; e Department of Human Movement Sciences, Faculty of Behavioral and Movement Sciences, Vrije Universiteit Amsterdam, Amsterdam Movement Sciences, Amsterdam, The Netherlands

**Keywords:** Artificial intelligence (AI), ChatGPT, Acromioclavicular dislocation, Patient education, Sports medicine, Internet

## Abstract

**Background:**

ChatGPT, an expanding artificial intelligence platform, is rapidly becoming a source of medical knowledge for patients. The purpose of this study is to evaluate the quality and readability of information provided by ChatGPT 4.0 in response to the most frequently asked patients' questions regarding acromioclavicular (AC) joint dislocations.

**Methods:**

Twenty-five frequently asked patient questions regarding AC joint dislocations were posed to ChatGPT 4.0. The quality and accuracy of the responses was graded by two fellowship-trained shoulder surgeons using a 5-point Likert scale. Responses were analyzed for readability using six established metrics.

**Results:**

ChatGPT provided responses to 25 frequently asked questions, with consensus reached on 12 questions. The final average score was 4/5, reflecting good quality with minor inaccuracies. Treatment-related questions scored lower compared to other categories, with a significant difference observed between treatment and rehabilitation (*P* = .025). Cohen's kappa indicated poor correlation (0.085), though the percent agreement was 48%, increasing to 100% when allowing for a 1-point difference. Readability scores revealed moderate difficulty levels, suitable for a high school-level audience.

**Conclusion:**

ChatGPT delivers accurate and easily comprehensible information on AC joint dislocations, highlighting its potential to improve patient education. Although the model generally provides high-quality responses, its limitations in addressing treatment-related questions underscore the importance of clinician oversight. ChatGPT can therefore serve as a valuable complement to traditional patient education methods.

Acromioclavicular (AC) joint dislocations are common shoulder injuries, typically resulting from trauma, direct impact to the shoulder, or fall on an outstretched hand.^18^ Significant controversy remains regarding proper diagnosis and management of AC joint dislocations. While health care professionals play an important role in the acute and ongoing care of these injuries, patient education is equally vital to ensure successful outcomes. Patient education is a cornerstone in delivering quality care and has been shown to be correlated with higher patient satisfaction postoperatively.^4^^,^^11^^,^^19^ Traditionally, patient education has depended on face-to-face consultations, written materials, and follow-up visits. However, with the rapid growth of artificial intelligence (AI) tools—especially large language models like ChatGPT (OpenAI, San Francisco, CA, USA)—there is now an innovative opportunity to enhance patient education and assist health care professionals in delivering essential information. Several studies in other medical specialties have explored the effectiveness of ChatGPT-generated responses to frequently asked questions (FAQs), yielding largely positive outcomes.^14^^,^^17^^,^^20^, ^21^, ^22^

This study aims to evaluate the utility of ChatGPT as a resource for providing accurate, accessible, and understandable information to patients with AC joint dislocations. We compared these metrics for questions within five domains, namely background, diagnosis, treatment, rehabilitation and outcome. By examining its effectiveness as both an informational tool for clinicians and a means of patient education, we explore the potential of AI in enhancing patient understanding of their condition, treatment options, and rehabilitation protocols. Furthermore, this paper discusses the limitations and challenges associated with relying on AI-driven platforms for patient education. Through this evaluation, we seek to inform future applications of AI for patient education, and thus improve the quality of care provided to individuals suffering from AC joint dislocations. We hypothesize that a commonly used AI driven platform (ChatGPT 4.0) will be able to provide high-quality and accurate information to patients and primary care clinicians about AC joint dislocations.

## Methods

**Figure 1:**
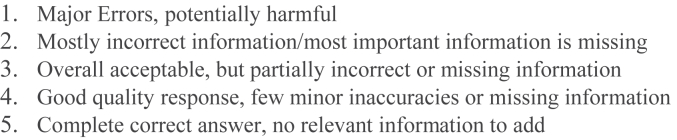
Five-point Likert scale for accuracy of responses.

Our study design was based on a methodology similar to those used in previously published orthopedic studies.^7^^,^^9^^,^^14^^,^^21^ Twenty-five FAQs regarding AC joint dislocations were assembled and reviewed by two fellowship-trained shoulder surgeons (MV and SG), using a 5-point Likert scale, as has been done in previous studies. The 5-point Likert scale utilized in the present study is displayed in Figure 1.^23^ The Likert scale was designed to evaluate general measures of accuracy in terms of content.

**Figure 2:**
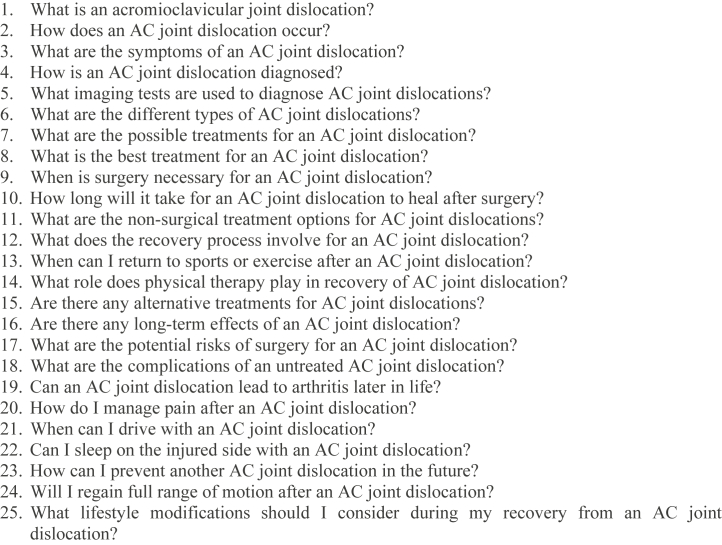
FAQs about AC joint dislocations. *FAQ*, frequently asked question; *AC*, acromioclavicular.

A list of FAQ’s were compiled from the websites of international orthopedic/shoulder societies (e.g. American Academy of Orthopaedic Surgeons) and major healthcare institutions (John Hopkins Medicine, King's College Hospital NHS, Mayo Clinic, Hospital for Special Surgery, The Steadman Clinic) and was screened in consultation with the senior author (MB).^6^^,^^8^^,^^10^^,^^12^^,^^25^ The twenty-five most pertinent questions related to common patient concerns about diagnosis, management, and rehabilitation related to AC joint dislocations were selected for accuracy analysis and reviewed by two fellowship-trained shoulder surgeons (MV and SG). Any disagreements in ratings were resolved by a senior author with subspecialty training in shoulder surgery (MB). The selected questions are displayed below in Figure 2. Each question was entered one by one into ChatGPT 4.0 (Open AI, San Francisco, CA, USA) to avoid altered responses due to prior question memory. No prompts were used prior to posing the questions in order to replicate a real-life situation as accurately as possible. No repeat or follow-up queries were asked. Responses were directly exported into a separate document (Google Forms; Alphabet Inc., Mountain View, CA, USA) without alteration.

Readability of each response was assessed by determining several established measures such as the Flesch Kincaid Reading Ease , Flesch Kincaid Grade Level, Gunning Fog score, SMOG index, Automated Readability index and Coleman-Liau Index. The Flesch Kincaid Reading Ease score correlates with the ease of reading content, with a score that ranges from “unreadable” at 0 to “easy to read” at 100. The six metrics above provide a comprehensive view of text complexity/readability.

### Statistical analysis

Statistical analysis was performed using Statistical Package for the Social Sciences Statistics (version 2; SPSS Inc., Chicago, IL, USA). Responses generated by Chat GPT 4.0 were assessed in terms of mean readability and quality by two fellowship-trained shoulder surgeons. Descriptive statistics were performed, including median, mode, and ranges, to describe ChatGPT's accuracy and readability for each question. Normally distributed continuous variables were reported in means and compared using independent t-tests. All analyses were two-tailed, and a *P* value of < .05 was considered statistically significant. Weighted Cohen's kappa coefficient test of inter-rater reliability was utilized to assess agreement between reviewers. Since both reviewers are well trained, percent agreement was calculated as well.^13^

## Results

The ChatGPT responses on the twenty-five FAQs can be found in Supplementary Appendix S1. The reviewers achieved consensus on 12 out of the 25 questions, with scores ranging from 3 to 5 on the 5-point Likert scale. The average scores were 4.3 for reviewer 1 and 4 for reviewer 2. After discrepancies were arbitrated by the senior author, the final overall score was 4 out of 5, indicating a good quality response, with only few minor inaccuracies or missing information. The different scores from each reviewer can be found in Table I.

**Table I:** Grades of ChatGPT responses to frequently asked questions regarding AC dislocations.

Question #	Question	Reviewer 1	Reviewer 2	Reviewer 3 (if necessary)	Final rating
1	What is an AC joint dislocation?	4	4	-	4
2	How does an AC joint dislocation occur?	4	4	-	4
3	What are the symptoms of an AC joint dislocation?	4	5	5	5
4	How is an AC joint dislocation diagnosed?	4	4	-	4
5	What imaging tests are used to diagnose AC joint dislocations?	4	4	-	4
6	What are the different types of AC joint dislocations?	4	3	4	4
7	What are the possible treatments for an AC joint dislocation?	4	3	3	3
8	What is the best treatment for an AC joint dislocation?	4	3	3	3
9	When is surgery necessary for an AC joint dislocation?	4	3	3	3
10	How long will it take for an AC joint dislocation to heal after surgery?	4	5	4	4
11	What are the nonsurgical treatment options for AC joint dislocations?	4	4	-	4
12	What does the recovery process involve for an AC joint dislocation?	4	4	-	4
13	When can I return to sports or exercise after an AC joint dislocation?	4	4	-	4
14	What role does physical therapy play in recovery of AC joint dislocation?	4	4	-	4
15	Are there any alternative treatments for AC joint dislocations?	5	4	4	4
16	Are there any long-term effects of an AC joint dislocation?	5	4	4	4
17	What are the potential risks of surgery for an AC joint dislocation?	4	5	4	4
18	What are the complications of an untreated AC joint dislocation?	5	4	4	4
19	Can an AC joint dislocation lead to arthritis later in life?	4	3	3	3
20	How do I manage pain after an AC joint dislocation?	4	4	-	4
21	When can I drive with an AC joint dislocation?	5	5	-	5
22	Can I sleep on the injured side with an AC joint dislocation?	5	4	4	4
23	How can I prevent another AC joint dislocation in the future?	5	4	4	4
24	Will I regain full range of motion after an AC joint dislocation?	5	5	-	5
25	What lifestyle modifications should I consider during my recovery from an AC joint dislocation?	4	4	-	4

*AC*, acromioclavicular.

**Figure 3:**
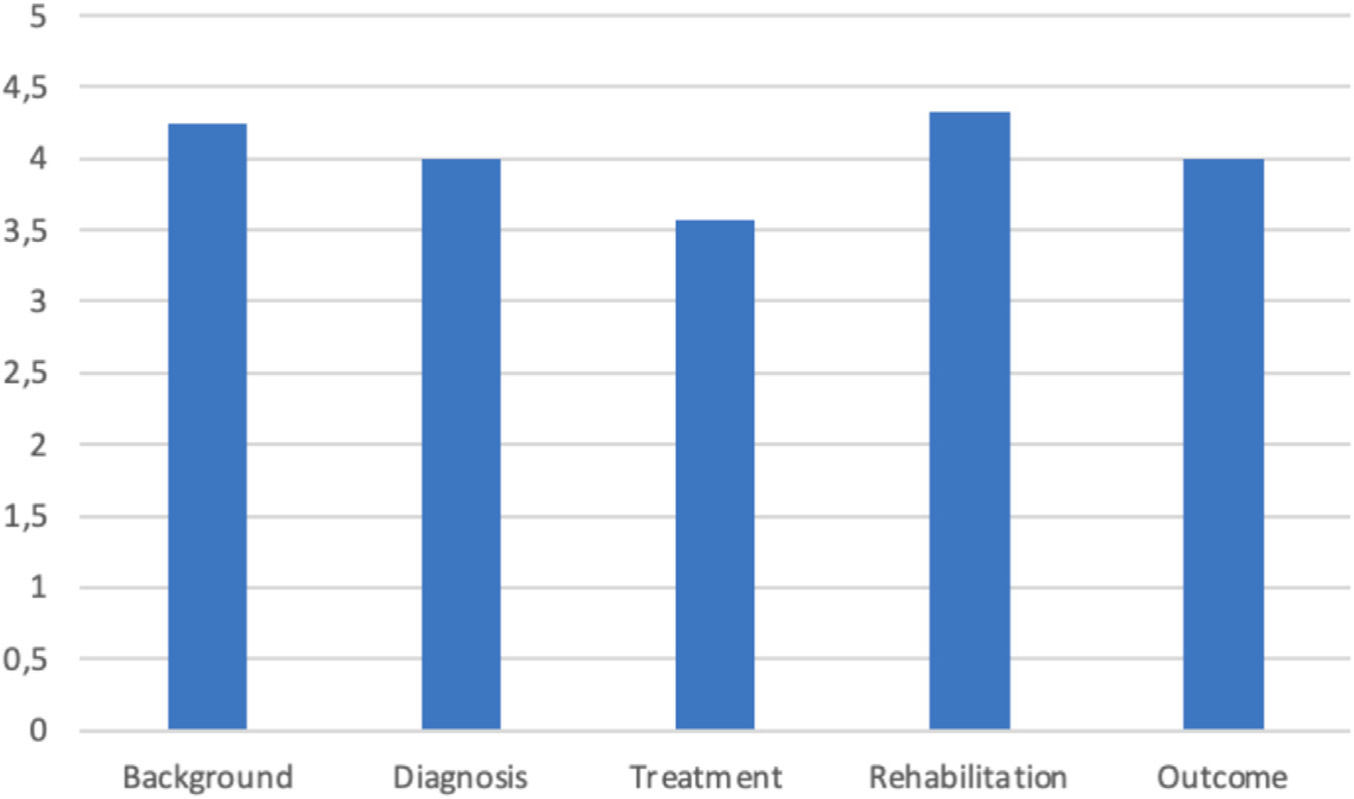
Question subtypes and their scores.

The questions were categorized into five groups: background, diagnosis, treatment, rehabilitation, and outcome. Scores for treatment-related questions were notably lower compared to those in other categories. The mean values for each category are presented in Figure 3. No significant differences were observed among the categories, with the exception of the comparison between treatment and rehabilitation, which showed a statistically significant difference (3.6 vs. 4.3, *P* = .025).

Cohen's kappa coefficient between the two reviewers was 0.085, indicating a poor correlation between the reviewers and a *P* value of .504. The percent agreement among reviewers was 48%. However, when considering both exact matches and scores differing by only 1 point, the agreement reached 100%.

The readability scores for each response generated by ChatGPT 4.0 are presented in Table II. In summary, based on the various readability measures calculated, ChatGPT 4.0 produced content written at a moderate difficulty level, appropriate for a high school audience. Given that AC joint dislocations are rarely seen in patients under 18, the responses generated by ChatGPT are suitable for most patients with this type of injury.^2^

**Table II:** Readability metrics.

Scoring system	Result	Meaning
Flesch Kincaid Reading Ease	39.9	Difficult
Flesch Kincaid Grade Level	11.9	Suitable for high school students
Gunning Fog Score	14.6	Requires more effort to read
SMOG Index	11.2	Moderate difficulty
Automated Readability Index	11.8	Standard for educated adults
Coleman-Liau Index	15.2	Requires higher education

*SMOG*, Simple Measure of Gobbledygook.

## Discussion

The integration of AI into health care has the potential to revolutionize patient education and clinical decision-making. This study sought to evaluate the quality of information provided by ChatGPT 4.0 in response to common patient questions regarding AC joint dislocations. Our findings demonstrate that ChatGPT 4.0 reliably provided high-quality and accurate information in response to research questions pertaining to AC joint dislocations, supporting our hypothesis. This suggests that AI-driven platforms like ChatGPT may serve as valuable adjuncts in patient education and clinical practice, particularly in conditions where clear communication of diagnostic, therapeutic, and rehabilitative information is critical.

While ChatGPT 4.0 demonstrated strong performance in providing accurate and high-quality information regarding the background, diagnosis, outcomes, and rehabilitation of AC joint dislocations, its responses to treatment-related questions, though still of high quality, were comparatively less robust and lacked complete information. We believe this discrepancy likely reflects the ongoing controversy and lack of consensus in literature regarding the optimal treatment strategies for different types of AC joint dislocations, rather than a deficiency in the platform's ability to deliver reliable information.^1^^,^^5^^,^^16^^,^^24^ Especially, the nonoperative treatment options for high-grade AC joint dislocations are either insufficiently addressed or not emphasized at all. While ChatGPT can serve as a valuable resource for patients and clinicians, its limitations in addressing treatment-related questions highlight the importance of clinician guidance. Treatment decisions for AC joint dislocations often require careful consideration of individual patient factors, such as activity level, functional demands, and personal preferences, which AI cannot fully assess or incorporate. Therefore, clinicians must continue to play a central role in guiding patients through these complex decisions, ensuring that care is tailored to their unique circumstances.

In addition, ChatGPT provides generalized and 1-dimensional responses to each question. This may give the impression that there is a single correct treatment for each grade of AC joint dislocation. When discussing complications, it is also essential to provide the percentage of cases in which they occur, as this can offer a more accurate perspective. This could lead to misunderstandings, with patients either fearing surgery unnecessarily or assuming that all high-grade dislocations should be treated surgically. Such oversimplifications highlight the need for health care professionals to engage in shared decision-making with their patients, emphasizing the importance of critical thinking and problem-solving skills.

The role of ChatGPT in patient education is increasingly significant and is expected to grow, particularly as younger patient populations, who are more frequently affected by AC joint dislocations, rely more on digital platforms for health information. These patients are shifting away from traditional sources, such as printed materials and in-person consultations, in favor of online resources. As health care professionals, it is our responsibility to safeguard patients from misinformation and ensure that AI-driven tools provide accurate, comprehensive, and evidence-based content. A theoretical advantage of ChatGPT-based education is that new information and evidence would be automatically incorporated, in contrast to traditional printed patient education brochures used in hospitals. This is especially important in the context of high-grade AC joint dislocations, where there is ongoing debate regarding treatment strategies for acute injuries and uncertainty surrounding the need for surgical intervention. It is crucial that patients have access to current, evidence-based information, empowering them to make informed decisions about their treatment in collaboration with their health care providers.

Another potential advantage of using ChatGPT in health care is its ability to improve productivity and enhance communication between doctors and patients. By providing patients with easily accessible, reliable information, ChatGPT allows them to become better informed about their conditions and treatment options before consultations. This leads to more focused and efficient discussions during appointments, as patients are already equipped with foundational knowledge. As a result, physicians can address more complex aspects of care, improving overall efficiency and potentially reducing consultation times without compromising the quality of care.

An overall score of 4 out of 5 for ChatGPT's responses indicates high overall quality with few minor inaccuracies or missing information. Cohen's kappa value was relatively low in this study, indicating limited inter-rater reliability. However, this might not fully capture the true level of agreement, as all scores were consistently within the range of 3-5, and no discrepancies exceeded 1 point. This is further supported by the percent agreement, which reached 100% when considering both exact matches and scores differing by only 1 point. In addition, percent agreement is considered a more reliable measure when raters are well trained, as in this study.^13^

The moderate readability level of ChatGPT's responses, appropriate for a high school audience, is a second interesting finding in this study. Since AC joint dislocations typically occur in young adults, responses tailored to a high school reading level are likely suitable for the majority of patients who experience this injury.^18^ Most individuals presenting with AC joint dislocations are well within the cognitive ability to comprehend information written at this level, making ChatGPT's readability both accessible and appropriate. Our findings contrast with previous orthopedic studies, which have shown that online patient educational resources on various topics, such as ulnar collateral ligament reconstruction, lateral epicondylitis, and shoulder arthroplasty, are often written at a level that exceeds the recommended reading comprehension level.^3^^,^^15^^,^^21^

### Limitations

Our study does have several limitations. There may be prevalent patient inquiries that have been omitted. Although ChatGPT is among the most popular AI programs, there are currently numerous other AI programs (eg, DeepSeek, Copilot, Perplexity, Google Search) that are also widely used. In this study, we focused solely on the English-language version of the ChatGPT responses and, as such, are unable to determine whether these findings are applicable to other languages. The process of selecting “top 25 FAQs” inherently involves a degree of subjectivity. The subjective nature of the assessment process poses additional challenges. The reliance on individual evaluators' judgments to rate the accuracy and quality of ChatGPT's responses introduces a level of variability that makes it more difficult to corroborate the findings universally. To our knowledge, this is the first study investigating the quality of AI-generated responses about AC joint dislocations.

## Conclusion

ChatGPT demonstrated the ability to provide accurate information about AC joint dislocations in response to patient queries and is written at an appropriate reading level for the average patient with an AC joint dislocation. It yields mostly good-quality responses, with only few minor inaccuracies or missing information responses to FAQs on AC joint dislocations. There is a great potential of this commercially available large language models like ChatGPT to enhance patient education of AC dislocations and other orthopedic conditions. However, its limitations in addressing treatment-related questions highlight the importance of clinician guidance and patients must be aware of these limitations. ChatGPT can thus play a supplementary role in patient education alongside traditional methods.

## Disclaimers:

Funding: No funding was disclosed by the authors.

Conflicts of interest: Michel van den Bekerom, MD, PhD reports grants for clinical and research fellowships supported by Smith & Nephew. The other authors have nothing to disclose. None of the fees above were related to the current study.
