# Supplementary material for: Appraisal of ChatGPT's responses to common patient questions regarding acromioclavicular joint dislocations
Source: JSES Rev Rep Tech. 2025 Aug 5;5(4):616–20. doi: 10.1016/j.xrrt.2025.06.020 (PMC12573632; doi:10.1016/j.xrrt.2025.06.020)
Supplement: Supplementary Appendix S1 [file mmc1.docx]

1. What is an acromioclavicular joint dislocation?

An acromioclavicular (AC) joint dislocation occurs when the acromioclavicular joint—the joint between the clavicle (collarbone) and the acromion (part of the shoulder blade)—becomes displaced or separated. This can happen as a result of trauma, usually from a fall onto the shoulder, a direct blow, or a forceful impact during sports or accidents.

Types of AC Joint Dislocations:

The severity of the injury is graded on a scale from Grade 1 to Grade 6:

- Grade 1: A mild sprain of the ligaments without significant displacement.
- Grade 2: A partial tear of the ligaments, leading to some displacement of the joint.
- Grade 3: A complete tear of the ligaments with a more noticeable displacement.
- Grade 4: The clavicle is displaced posteriorly (toward the back).
- Grade 5: Severe displacement, with the clavicle pushed up above the acromion.
- Grade 6: Rare, involving the clavicle being displaced under the acromion.

Symptoms:

- Pain at the top of the shoulder.
- Swelling and bruising around the joint.
- Limited range of motion in the shoulder.
- Visible deformity in severe cases, such as the "bump" or "step-off" deformity, where the clavicle is visibly raised.
- Tenderness when touching the joint area.

Treatment:

- Non-surgical (for mild cases): Rest, ice, anti-inflammatory medications, physical therapy, and possibly a sling to stabilize the shoulder.
- Surgical (for severe cases, like Grade 3 or higher): Surgery may be required to realign the joint and repair the ligaments.

AC joint dislocations can vary in severity, and the treatment will depend on the grade of the injury and the individual’s activity level. Most cases recover well with proper care.

1. How does an AC joint dislocation occur?

An **AC joint (Acromioclavicular joint) dislocation** occurs when there is an injury to the joint where the **clavicle (collarbone)** meets the **acromion** (the highest point of the shoulder blade). This dislocation is commonly caused by a **traumatic impact** or force that disrupts the normal alignment of the joint.

Here are some common ways an AC joint dislocation can happen:

1. **Direct impact or blow to the shoulder**: This is the most common cause. For example, falling directly onto the shoulder or being hit in a contact sport like football or rugby. The force of the impact pushes the acromion downward while the clavicle is forced upward, stretching or tearing the ligaments that hold the joint together.
2. **Falling on an outstretched arm**: A fall onto the hand or arm can transfer the force to the shoulder joint, resulting in an AC joint dislocation. The force travels through the arm to the shoulder, often causing the joint to separate.
3. **High-impact sports**: Activities that involve contact or heavy physical exertion, such as wrestling, skiing, cycling, or hockey, can increase the risk of an AC joint dislocation.
4. **Repetitive stress or overuse**: Although less common, repeated overuse or stress on the shoulder, such as from lifting heavy weights or other activities, can strain the AC joint and make it more vulnerable to dislocations over time.

In an AC joint dislocation, the severity can vary, ranging from a mild sprain of the ligaments (Grade 1) to complete tearing of the ligaments and a visible separation between the acromion and the clavicle (Grade 3 or higher). Treatment depends on the grade of dislocation, but it can include rest, physical therapy, and in more severe cases, surgery.

1. What are the symptoms of an AC joint dislocation?

An AC (acromioclavicular) joint dislocation occurs when the collarbone (clavicle) is separated from the shoulder blade (scapula) at the AC joint. This injury is common in sports, especially those involving contact or falls. The symptoms can vary depending on the severity of the dislocation but typically include:

1. **Pain at the top of the shoulder**: The most noticeable symptom, often sharp and localized around the AC joint.
2. **Swelling**: Swelling may develop at the site of the dislocation.
3. **Bruising**: Discoloration can appear around the shoulder area.
4. **Deformity**: A visible bump or protrusion may appear at the top of the shoulder, as the collarbone may rise due to the displacement.
5. **Limited Range of Motion**: Moving the arm, especially overhead or across the body, can be painful or difficult.
6. **Tenderness**: The area around the joint may be sensitive to touch.
7. **Instability**: You might feel a sensation of the shoulder being "loose" or unstable.
8. **Weakness**: Difficulty lifting or carrying things with the affected arm due to pain and weakness.

In severe cases, the dislocation can involve tearing of the ligaments that stabilize the joint, and additional symptoms may include significant pain and an inability to move the arm.

If you suspect an AC joint dislocation, it's important to seek medical attention for proper diagnosis and treatment.

1. How is an AC joint dislocation diagnosed?

An AC (acromioclavicular) joint dislocation is typically diagnosed through a combination of the following methods:

**1. Physical Examination:**

- **Inspection:** The doctor may look for visible deformities or swelling around the shoulder. In cases of significant dislocation, there may be an obvious bump or deformity due to the displaced collarbone.
- **Palpation:** The physician will gently press around the AC joint to check for tenderness and misalignment.
- **Range of Motion Tests:** The doctor will ask the patient to move their arm in different directions to assess the severity of the injury, pain levels, and any restricted movement.
- **Special Tests:**
  - **Cross-Body Adduction Test:** The patient brings their arm across the body, and pain or discomfort in the AC joint area could indicate a dislocation.
  - **Piano Key Sign:** The doctor presses down on the distal end of the clavicle, and if it moves up and down like a piano key, it may indicate an AC joint injury.

**2. Imaging:**

- **X-Rays:** The most common imaging tool for diagnosing an AC joint dislocation. The doctor will often request X-rays in multiple views, including an **AP (anteroposterior)** view and a **Zanca view** (a special view that helps show the AC joint more clearly).
- **Stress X-ray:** In some cases, the doctor may apply a gentle force to the shoulder to see how the joint moves under stress. This can help determine the severity of the dislocation and whether there's any ligament damage.
- **MRI or CT Scan:** These may be used for more complex or severe cases to evaluate associated soft tissue injuries, such as tears in the ligaments or cartilage damage.

These diagnostic methods help to classify the dislocation into different grades, from mild to severe, and guide treatment decisions.

1. What imaging tests are used to diagnose AC joint dislocations?

To diagnose an acromioclavicular (AC) joint dislocation, several imaging tests can be used:

1. **X-ray**:
   - This is the most common and first-line imaging test. It helps assess the alignment of the bones in the AC joint. X-rays can reveal whether the clavicle is displaced relative to the acromion. For better clarity, sometimes specific views like the **Zanca view** (an angled X-ray of the shoulder) are used.
2. **CT scan (Computed Tomography)**:
   - A CT scan provides detailed images of the bones and can be particularly helpful in assessing complex or severe dislocations, fractures associated with the AC joint, or if the X-ray results are unclear.
3. **MRI (Magnetic Resonance Imaging)**:
   - MRI is useful to evaluate soft tissues like ligaments and tendons. It can reveal damage to the ligaments that stabilize the AC joint, such as the **coracoclavicular ligament**, which is important in assessing the severity of the dislocation and potential for surgical intervention.

In general, an X-ray is the first test ordered to confirm the diagnosis, with CT or MRI used for more detailed evaluation if necessary.

1. What are the different types of AC joint dislocations?

The **Acromioclavicular (AC) joint** is where the collarbone (clavicle) meets the highest point of the shoulder blade (acromion). AC joint dislocations are common injuries, typically caused by trauma or falls. There are several types of AC joint dislocations, classified by the **Rockwood classification**, which categorizes them based on the severity of the injury and how much the clavicle has been displaced.

**1. Type I (Mild Sprain)**

- **Description:** This is the least severe form of AC joint injury. The ligament holding the clavicle to the acromion (the acromioclavicular ligament) is stretched or partially torn, but there is no significant displacement of the bones.
- **Symptoms:** Mild pain, swelling, and tenderness over the joint, but no noticeable deformity.
- **Treatment:** Rest, ice, anti-inflammatory medications, and physical therapy.

**2. Type II (AC Ligament Torn)**

- **Description:** In a Type II dislocation, the acromioclavicular ligament is completely torn, but the coracoclavicular (CC) ligaments (which help stabilize the clavicle) remain intact. The clavicle may shift upward slightly, but the displacement is not severe.
- **Symptoms:** More noticeable pain and swelling, along with some visible deformity or bump in the shoulder area.
- **Treatment:** Rest, ice, and physical therapy, but more significant injuries may require surgery.

**3. Type III (Complete Dislocation)**

- **Description:** Both the acromioclavicular and coracoclavicular ligaments are torn, leading to a more significant displacement of the clavicle (the end of the clavicle may rise above the acromion). This is a more severe injury.
- **Symptoms:** Severe pain, visible deformity, and difficulty moving the arm.
- **Treatment:** Surgery is often recommended to restore the alignment and stability of the shoulder joint, especially in athletes or individuals with high functional demands.

**4. Type IV (Posterior Dislocation)**

- **Description:** In a Type IV dislocation, the clavicle is displaced posteriorly (towards the back of the body) and may push into the muscles and tissues at the back of the shoulder. This is rare and can be quite serious.
- **Symptoms:** Severe pain, difficulty moving the arm, and a significant deformity. It may also cause compression of nerves or blood vessels.
- **Treatment:** Surgery is generally required to reposition the clavicle and repair the ligaments.

**5. Type V (Severe Dislocation)**

- **Description:** This is the most severe form of AC joint dislocation. The clavicle is displaced far superiorly (above the acromion), often more than Type III dislocations. Both the acromioclavicular and coracoclavicular ligaments are torn, and there is significant displacement of the clavicle.
- **Symptoms:** Significant deformity, severe pain, and functional loss. The shoulder appears visibly abnormal with a "step-off" between the clavicle and acromion.
- **Treatment:** Surgery is often required to repair the joint and restore shoulder function.

**6. Type VI (Inferior Dislocation)**

- **Description:** A very rare and severe type of dislocation where the clavicle is displaced inferiorly (below the acromion), usually into the subacromial or subcoracoid space. It is often associated with other severe injuries.
- **Symptoms:** Severe pain, deformity, and difficulty moving the shoulder, along with other injuries depending on the direction of displacement.
- **Treatment:** Surgery is usually required to restore normal anatomy and function.

**Treatment Overview:**

- **Conservative (Non-Surgical) Treatment:** For Type I and Type II dislocations, non-surgical management such as rest, ice, and physical therapy is often effective.
- **Surgical Treatment:** More severe dislocations (Type III and above) may require surgery, especially if there is a significant deformity or if the patient is active and needs to restore full function.

In all cases, a healthcare provider will determine the best treatment based on the severity of the injury and the patient's individual needs.

1. What are the possible treatments for an AC joint dislocation?

An acromioclavicular (AC) joint dislocation, which occurs when the collarbone (clavicle) and the shoulder blade (scapula) separate, is a common injury, especially in athletes or people involved in contact sports. The treatment depends on the severity of the dislocation, which is typically graded from I to VI.

Here are the treatment options based on the severity of the dislocation:

**1. Grade I (Mild Sprain):**

- **Rest and Ice**: Applying ice to reduce swelling and taking rest from activities that put strain on the shoulder.
- **Pain Management**: Over-the-counter pain medications like ibuprofen or acetaminophen can be used to manage pain.
- **Physical Therapy**: Once the acute pain subsides, physical therapy to improve strength and mobility of the shoulder joint may be recommended.
- **Shoulder Sling**: In some cases, wearing a sling may provide additional support for a short period.

**2. Grade II (Partial Dislocation):**

- **Rest and Ice**: Like in Grade I, rest and ice are often recommended.
- **Pain Management**: Medications may be used to manage pain and inflammation.
- **Shoulder Sling or Immobilization**: A sling or brace may be worn for a few weeks to immobilize the shoulder.
- **Physical Therapy**: After initial healing, physical therapy is essential to regain range of motion and strength in the shoulder.

**3. Grade III (Complete Dislocation with Separation):**

- **Non-Surgical Treatment**: In some cases, nonsurgical management may be sufficient, especially if the separation is mild or the patient is not engaged in heavy physical activities. Treatment includes:
  - Rest, ice, and medications to manage pain and swelling.
  - Sling or brace for immobilization.
  - Physical therapy post-healing.
- **Surgical Treatment**: Surgery may be recommended for athletes or individuals with high demands on shoulder function. Surgical options may involve:
  - **AC Joint Reconstruction**: Repairing or reconstructing the ligaments that stabilize the AC joint.
  - **Ligament Repair or Grafting**: In severe cases, ligaments may need to be repaired or replaced with grafts from another part of the body.

**4. Grade IV, V, and VI (Severe Dislocations):**

- **Surgical Intervention**: These grades typically require surgery to correct the dislocation. The goal of surgery is to realign the bones and stabilize the joint. Surgical options may include:
  - **AC Joint Reduction**: Repositioning the collarbone and shoulder blade to their proper anatomical alignment.
  - **Ligament Reconstruction**: If ligaments are severely damaged, they may be reconstructed using grafts.
  - **Clavicle Fixation**: In some cases, a plate and screws may be used to secure the clavicle in place.

**Post-Treatment Considerations:**

- **Rehabilitation**: Regardless of the treatment method, rehabilitation is crucial to restore full function and strength. Physical therapy typically starts with gentle range-of-motion exercises and progresses to strengthening exercises.
- **Surgery Recovery**: After surgery, the recovery period might take several months, and full healing can take 6 to 12 months depending on the severity of the injury and the type of surgery performed.

It’s important to consult an orthopedic specialist to determine the most appropriate treatment based on the specific grade and individual needs.

1. What is the best treatment for an AC joint dislocation?

The treatment for an AC (acromioclavicular) joint dislocation can vary based on the severity of the injury (grade 1, 2, 3, etc.) and the specific circumstances of the patient. Here's an overview of common treatment options:

**Conservative (Non-Surgical) Treatment:**

For **mild** cases (Grade 1 or 2) where the joint is not displaced too far, conservative treatment is often recommended:

1. **Rest**: Avoid activities that stress the shoulder, especially overhead movements.
2. **Ice**: Apply ice to reduce swelling and inflammation, typically in the first 48 hours after injury.
3. **Pain Management**: Over-the-counter medications (e.g., ibuprofen, acetaminophen) to control pain and inflammation.
4. **Sling or Brace**: A shoulder sling may be used for comfort to immobilize the shoulder while healing.
5. **Physical Therapy**: Once the pain and swelling subside, strengthening exercises and range-of-motion exercises will help restore shoulder function and prevent stiffness.

**Surgical Treatment:**

For **more severe** cases (Grade 3, 4, 5, or 6), where the joint is significantly displaced or there is damage to ligaments, surgery may be required. Surgical options include:

1. **AC Joint Reconstruction**: Involves repairing or reconstructing the ligaments around the joint, often using tendon grafts or synthetic material to stabilize the area.
2. **AC Joint Resection**: Involves removing the distal part of the clavicle to reduce pain and allow for more mobility.
3. **Pins/Screws**: Sometimes a surgeon may use pins, screws, or plates to hold the bones in place while they heal.

**Post-Treatment Care:**

- **Rehabilitation**: Whether conservative or surgical treatment is used, physical therapy plays a crucial role in restoring strength, range of motion, and function to the shoulder.
- **Gradual Return to Activity**: Depending on the severity of the injury, you may need to gradually ease back into your normal activities and avoid high-impact or contact sports until fully healed.

**When to Seek Medical Attention:**

- **Severe pain**
- **Noticeable deformity** or dislocation of the joint
- **Inability to move the shoulder**
- **Numbness or tingling** in the arm or hand (could indicate nerve involvement)

If you're unsure about the degree of the dislocation or the best course of treatment, seeing a healthcare professional is important for an accurate diagnosis and tailored treatment plan.

1. When is surgery necessary for an AC joint dislocation?

Surgery for an AC (acromioclavicular) joint dislocation is generally considered when the injury is more severe, or if conservative treatments (like rest, ice, and physical therapy) don’t provide relief or fail to restore normal function. Here are some circumstances in which surgery might be necessary:

1. **Grade 3, 4, or 5 AC Joint Dislocations**: These are more severe dislocations where the ligaments that stabilize the joint are completely torn. In these cases, the collarbone may be significantly displaced, and surgery is often needed to realign the joint and secure it in place.
2. **Failure of Conservative Treatment**: If non-surgical treatments (like physical therapy, rest, and anti-inflammatory medications) don’t relieve pain or improve function, surgery might be recommended.
3. **Persistent Pain and Dysfunction**: If there is ongoing pain and the dislocation is interfering with daily activities, sports, or work, surgery may be necessary to stabilize the joint and allow for better movement.
4. **Younger, Active Patients**: In young and active individuals, surgery may be recommended to restore the joint to its normal function, especially if they want to return to sports or physically demanding activities.
5. **Severe Deformity**: If the dislocation leads to a visible deformity (e.g., a "bump" on the shoulder that doesn't resolve), surgery may be considered to correct the alignment and prevent long-term issues.
6. **Complications or Associated Injuries**: If the dislocation is associated with fractures or other injuries to nearby structures (like the collarbone or shoulder blade), surgery may be required to address those as well.

Each case is unique, so it's important to discuss with a doctor or orthopedic specialist to determine the best approach. They will assess the specific type of dislocation, your overall health, and your activity level when recommending treatment.

1. How long will it take for an AC joint dislocation to heal after surgery?

After surgery for an AC (acromioclavicular) joint dislocation, the healing process can vary depending on the severity of the injury, the type of surgery performed, and the individual's health and rehabilitation efforts. On average, it typically takes around **6 to 12 weeks** for the initial healing, but full recovery and return to normal activities may take longer, around **4 to 6 months**.

Here's a general timeline:

- **First 2 weeks:** Rest and immobilization, with a sling to protect the shoulder.
- **Weeks 2–6:** Gradual range-of-motion exercises and physical therapy, but still avoiding heavy lifting or overhead activities.
- **6–12 weeks:** Increased strengthening exercises, but still cautious of the healing joint.
- **3–6 months:** Full recovery with the possibility of returning to sports and strenuous activity, depending on the healing progress.

It's important to follow the surgeon's instructions and work closely with a physical therapist to regain strength and mobility in the shoulder. Recovery times can differ based on the individual, so it’s always best to consult with your healthcare provider for personalized guidance.

1. What are the non-surgical treatment options for AC joint dislocations?

Non-surgical treatments for an acromioclavicular (AC) joint dislocation focus on reducing pain, controlling inflammation, and promoting healing. The approach depends on the severity of the dislocation. Here are some common non-surgical treatment options:

1. **Rest and Activity Modification**:
   - Avoid activities that put strain on the shoulder, particularly overhead movements or heavy lifting. Rest helps reduce strain and allows the healing process to begin.
2. **Ice Therapy**:
   - Applying ice to the injured area in intervals (15-20 minutes every 1-2 hours) can help reduce swelling and manage pain during the acute phase.
3. **Pain Medications**:
   - Nonsteroidal anti-inflammatory drugs (NSAIDs), like ibuprofen or naproxen, can help reduce pain and inflammation. Over-the-counter pain relievers are typically used for mild to moderate pain.
4. **Physical Therapy**:
   - Once the pain has decreased, physical therapy is important for restoring the range of motion, strength, and stability of the shoulder. Exercises may include stretches and strengthening routines to promote shoulder function and prevent future issues.
5. **Bracing or Sling**:
   - In some cases, a brace or sling is used to immobilize the shoulder and relieve pressure on the AC joint, which aids in healing. This can be particularly helpful in the early stages after the injury.
6. **Taping**:
   - In some cases, kinesiology tape or other taping methods can be applied to support the joint and reduce discomfort during movement.
7. **Steroid Injections**:
   - If pain persists, corticosteroid injections may be considered to reduce inflammation in the AC joint. This can provide relief, but it is generally a short-term solution.
8. **Gradual Return to Activity**:
   - Once healing progresses, a gradual return to normal activities is important. This involves focusing on strengthening exercises to support the shoulder and prevent re-injury.

In cases of mild to moderate dislocations (Grade 1 or 2), non-surgical treatment is typically sufficient. However, for more severe cases (Grade 3 or higher), surgery may be required if non-surgical methods do not lead to significant improvement. Always consult with a healthcare professional to determine the best course of treatment based on the specific injury.

1. What does the recovery process involve for an AC joint dislocation?

The recovery process for an **AC (acromioclavicular) joint dislocation** typically involves several stages, depending on the severity of the injury (graded from Type I to Type VI). Here's a general outline of what the recovery may involve:

**1. Initial Treatment (First 48-72 hours)**

- **Rest**: Avoid movements that stress the shoulder.
- **Ice**: Apply ice packs to the affected area to reduce swelling and pain.
- **Elevation**: If possible, elevate the arm to reduce swelling.
- **Pain management**: Over-the-counter pain medications (e.g., ibuprofen or acetaminophen) can help manage pain and inflammation.

**2. Immobilization**

- **Sling or brace**: In the first few days or weeks, a sling or brace may be used to stabilize the shoulder and restrict movement while the AC joint heals.
- **Duration of immobilization**: Depending on the injury severity, this might last anywhere from a few days to a few weeks.

**3. Rehabilitation (After initial healing phase)**

- **Range of motion exercises**: Once the pain begins to subside, you can start with gentle range-of-motion exercises to avoid stiffness.
- **Strengthening exercises**: After regaining full range of motion, strengthening exercises for the shoulder muscles (rotator cuff, deltoid) will help restore strength and stability.
- **Gradual return to activity**: Depending on the injury grade, return to normal activities (especially sports) is gradual to avoid re-injury.

**4. Physical Therapy**

- **Stretching and strengthening**: A physical therapist may guide you through specific exercises to strengthen the shoulder and improve mobility.
- **Proprioception training**: Focused exercises that help improve coordination and stability of the shoulder joint.

**5. Surgical Intervention (if necessary)**

- For more severe cases (typically higher-grade dislocations), surgery may be required. This might involve:
  - **AC joint stabilization**: Using screws, plates, or other techniques to restore the alignment of the bones.
  - **Ligament repair**: If ligaments are severely damaged, surgery may involve repairing or reconstructing them.

**6. Recovery Time**

- For **mild dislocations (Type I and II)**, recovery might take a few weeks to a couple of months.
- For **severe dislocations (Type III and higher)**, it may take several months, especially if surgery is required.
- The timeline also varies based on the individual’s age, fitness level, and adherence to rehabilitation protocols.

**7. Return to Sports/Physical Activities**

- Most people can return to non-contact activities within 6-12 weeks.
- Contact sports (e.g., football) may require a longer recovery period, sometimes up to 6 months, depending on the severity of the injury and how well the healing progresses.

Throughout the recovery process, it is important to follow medical advice, avoid activities that could reinjure the shoulder, and ensure that rehabilitation exercises are done correctly.

1. When can I return to sports or exercise after an AC joint dislocation?

Returning to sports or exercise after an AC (Acromioclavicular) joint dislocation can vary depending on the severity of the injury, your specific recovery, and your doctor’s advice. Here's a general timeline:

1. **Mild AC Joint Dislocation (Grade 1):**
   - **Recovery Time**: Typically 2-6 weeks.
   - **Return to Activity**: After symptoms improve (pain, swelling, and limited range of motion decrease), you can usually return to light activity, but avoid contact sports or overhead movements until fully healed.
2. **Moderate AC Joint Dislocation (Grade 2):**
   - **Recovery Time**: Around 4-8 weeks.
   - **Return to Activity**: After the pain subsides and range of motion is restored, you may begin low-impact exercises. It's important to ease into more strenuous activity slowly.
3. **Severe AC Joint Dislocation (Grade 3 or higher):**
   - **Recovery Time**: 8-12 weeks or more, depending on whether surgery was required.
   - **Return to Activity**: After surgery or conservative treatment, you'll need physical therapy to restore strength and stability. Contact sports may not be safe until you have regained full shoulder function and strength.

**General Guidelines:**

- **Follow-up Care**: Always check with your healthcare provider before returning to sports, as they can assess your healing and provide specific advice.
- **Rehabilitation**: A physical therapy program is essential to regain shoulder mobility and strength before resuming high-impact or overhead activities.
- **Pain and Swelling**: Avoid returning to activity if you're still experiencing significant pain or swelling.

Listen to your body, and make sure to progress gradually to prevent reinjury.

1. What role does physical therapy play in recovery of AC joint dislocation?

Physical therapy (PT) plays a crucial role in the recovery process following an acromioclavicular (AC) joint dislocation. The AC joint, located where the clavicle (collarbone) meets the acromion (a bony projection of the scapula), is involved in shoulder movement and stability. When the joint is dislocated, the ligaments connecting the bones can stretch or tear, resulting in pain, instability, and difficulty moving the shoulder. PT helps address these issues through several key stages of recovery:

**1. Pain Management and Initial Healing (Phase 1)**

- **Modalities for pain relief**: Techniques such as ice therapy, electrical stimulation, or ultrasound may be used to reduce pain and inflammation in the initial stages of recovery.
- **Protection of the joint**: In the early phase, PT focuses on protecting the AC joint while allowing soft tissue healing. This might include wearing a sling or brace and limiting motion to prevent further injury.

**2. Restoration of Range of Motion (Phase 2)**

- **Gentle stretching**: Once acute pain subsides, PT focuses on restoring the normal range of motion (ROM) in the shoulder. This involves gentle stretching exercises to gradually increase flexibility without overloading the joint.
- **Active-assisted exercises**: Initially, the therapist may guide the shoulder through passive or active-assisted movements to prevent stiffness and improve flexibility.

**3. Strengthening (Phase 3)**

- **Targeted strengthening**: Strengthening exercises for the rotator cuff muscles, deltoid, and scapular stabilizers are vital to regain shoulder stability and prevent future injuries. These exercises help stabilize the joint by improving muscle support around the AC joint.
- **Progressive load**: As healing progresses, exercises will gradually become more challenging, working toward more dynamic movements, such as overhead motions, with resistance bands or weights.

**4. Proprioception and Functional Training (Phase 4)**

- **Proprioception**: This is the sense of the body’s position in space. PT includes exercises designed to improve proprioception and balance, which helps to prevent re-injury. This often involves exercises that challenge shoulder stability and coordination.
- **Return to activity**: As strength and range of motion improve, PT prepares the patient for a return to normal activities, including sports or heavy lifting. Sport-specific rehabilitation and functional exercises might be incorporated.

**5. Prevention of Future Injuries**

- **Posture correction and biomechanics**: PT may also focus on improving posture and biomechanics to ensure the shoulder moves properly during daily activities and sports, reducing the risk of future dislocations.

Overall, physical therapy not only aids in reducing pain and improving function but also helps ensure the joint heals in the proper position, minimizing the risk of long-term issues like instability or arthritis. The specific exercises and techniques used depend on the severity of the dislocation and the individual's recovery progress, so PT is highly personalized.

1. Are there any alternative treatments for AC joint dislocations?

Yes, there are several alternative treatments for **AC (acromioclavicular) joint dislocations**, though the treatment approach depends on the severity of the injury. For milder dislocations (Grade I and II), non-surgical options are often effective, while more severe dislocations (Grade III and above) may require surgery. Here are some alternative treatments commonly used for less severe cases:

**1. Rest and Ice**

- **Rest**: Avoid activities that aggravate the shoulder, especially lifting or overhead movements.
- **Ice**: Applying ice packs for 20-30 minutes several times a day can reduce swelling and pain during the acute phase.

**2. Physical Therapy**

- A physical therapist can guide you through exercises to strengthen the shoulder muscles, improve mobility, and enhance the stability of the AC joint. Therapy may begin with gentle range-of-motion exercises and progress to strengthening exercises for the rotator cuff and scapular stabilizing muscles.
- **Taping** or **bracing** may also be used to provide additional support during rehabilitation.

**3. Non-steroidal Anti-inflammatory Drugs (NSAIDs)**

- NSAIDs like ibuprofen or naproxen can help manage pain and reduce inflammation around the AC joint.

**4. Sling or Shoulder Immobilizer**

- For moderate AC joint dislocations, wearing a sling or shoulder immobilizer for a few weeks can allow the joint to rest and heal. This helps in preventing excessive movement of the shoulder and reduces strain on the injured area.

**5. Manual Therapy**

- In some cases, a chiropractor or osteopath may use manual therapy techniques, such as joint mobilization or manipulation, to help restore movement and reduce pain.

**6. Corticosteroid Injections**

- If the pain is persistent and significant, a doctor might administer corticosteroid injections to reduce inflammation and pain in the joint. This is often used in cases where conservative measures haven't provided relief.

**7. Regenerative Medicine (Platelet-Rich Plasma, PRP)**

- PRP injections involve using a concentrated sample of your own platelets to promote healing of damaged tissues. This treatment is still under investigation for AC joint injuries but is an option some athletes or individuals explore for pain management and healing.

**8. Alternative Therapies (e.g., Acupuncture)**

- Some people use acupuncture as a complementary treatment for pain management, though it may not directly address the dislocation but can potentially alleviate discomfort.

**9. Restoration of Range of Motion**

- After the acute pain subsides, it’s important to gently work on regaining full shoulder range of motion to avoid stiffness and long-term dysfunction.

These approaches are generally aimed at **conservative management** and are most effective for **less severe dislocations** (Grades I and II). In more severe cases, particularly **Grade III** or higher, surgical intervention (such as **AC joint reconstruction**) may be required if conservative methods fail or if there's significant deformity or instability in the joint.

Would you like more details about any specific treatment, or are you currently dealing with an AC joint dislocation?

1. Are there any long-term effects of an AC joint dislocation?

Yes, there can be long-term effects following an acromioclavicular (AC) joint dislocation, although the severity of these effects can vary depending on the extent of the injury, treatment, and individual factors. Some of the potential long-term effects include:

1. **Chronic Pain**: Some people experience persistent pain or discomfort in the shoulder, particularly when performing overhead activities or lifting heavy objects. This pain may come from the joint itself or surrounding tissues that were affected by the injury.
2. **Arthritis**: Over time, the joint may develop arthritis, as the cartilage can be damaged during the dislocation. This can lead to increased wear and tear in the joint, causing stiffness, pain, and decreased range of motion.
3. **Instability**: Depending on the severity of the dislocation, the AC joint may become unstable. This can result in the shoulder feeling weak or prone to further dislocations, particularly with certain movements or activities that stress the joint.
4. **Decreased Range of Motion**: The injury and subsequent healing process can lead to stiffness in the shoulder, limiting its range of motion. This can make it difficult to fully raise the arm or perform specific movements, especially overhead.
5. **Cosmetic Changes**: A common result of a severe AC joint dislocation is the development of a "bump" or deformity in the shoulder, where the collarbone (clavicle) sticks up due to the separation of the joint. This might be visible and cause cosmetic concerns.
6. **Muscle Weakness**: The muscles around the shoulder may weaken due to prolonged immobility or disuse after the injury. This can contribute to further functional limitations and difficulty with activities that require shoulder strength.

Treatment plays a significant role in determining the long-term outcome. Many AC joint dislocations, especially mild to moderate ones, heal well with conservative management (rest, physical therapy, and anti-inflammatory treatments). However, more severe cases may require surgical intervention to restore joint stability and prevent long-term complications.

If you've had an AC joint dislocation or are experiencing persistent symptoms, working with a healthcare provider or orthopedic specialist is key to managing and reducing long-term effects.

1. What are the potential risks of surgery for an AC joint dislocation?

Surgery for an acromioclavicular (AC) joint dislocation, while often effective in restoring function and stability to the shoulder, carries some potential risks and complications. These include:

1. **Infection**: As with any surgery, there's a risk of infection at the incision site or deeper, which may require antibiotics or additional surgery to treat.
2. **Nerve Injury**: There's a risk of damaging nerves near the shoulder during surgery, which can lead to numbness, tingling, weakness, or even paralysis in severe cases.
3. **Blood Vessel Damage**: The surgery could inadvertently damage blood vessels, which could cause bleeding or clotting issues.
4. **Failure of Fixation**: The surgical fixation (e.g., using screws, plates, or ligament repair) might fail, leading to re-dislocation or instability of the joint. This could require additional surgery.
5. **Stiffness and Limited Range of Motion**: Post-surgery, some people experience limited shoulder mobility or stiffness, which can affect function and quality of life.
6. **Pain**: Chronic pain after surgery can occur, especially if the joint does not heal properly or if arthritis develops in the AC joint over time.
7. **Delays in Healing**: Sometimes, the bones may not heal as expected, leading to delayed or incomplete healing, which might necessitate further treatments.
8. **Clavicle Fracture**: In rare cases, hardware used to stabilize the joint might lead to a fracture of the clavicle (collarbone).
9. **Scar Tissue Formation**: Scar tissue can develop in and around the joint, potentially leading to restrictions in motion and discomfort.

Recovery from surgery typically involves physical therapy to help restore function, and the risks of surgery should be weighed against the potential benefits of improved shoulder stability and function. Discussing these with a surgeon will help ensure you understand the procedure and what to expect.

1. What are the complications of an untreated AC joint dislocation?

An untreated acromioclavicular (AC) joint dislocation can lead to several complications over time. Some of the key issues include:

1. **Chronic Pain**: If the injury isn’t treated, it may lead to ongoing discomfort in the shoulder, especially with overhead movements or lifting. This pain can become a long-term problem, limiting the use of the arm.
2. **Arthritis**: Over time, the joint may develop osteoarthritis, especially if the dislocation disrupts the cartilage or causes abnormal wear on the joint surfaces. This leads to stiffness, pain, and reduced mobility.
3. **Shoulder Instability**: The dislocation can cause the joint to remain unstable, leading to a higher risk of further dislocations or subluxations (partial dislocations). This instability can impair shoulder function and cause frequent episodes of the shoulder "giving way."
4. **Deformity**: A severe, untreated AC joint dislocation may result in visible deformity. The collarbone may protrude noticeably, often referred to as a "step deformity," where there is a bump at the top of the shoulder.
5. **Muscle Weakness**: With a dislocated AC joint, the surrounding muscles, especially the rotator cuff and trapezius, may become weakened due to reduced use or altered mechanics, leading to further loss of shoulder strength and function.
6. **Limited Range of Motion**: Scar tissue may form around the joint, and the injury could lead to a reduction in the shoulder's range of motion, making it difficult to perform certain activities, especially those that require full arm elevation or rotation.
7. **Tendon or Ligament Damage**: If the dislocation isn't treated, there may be prolonged damage to the ligaments and tendons surrounding the joint, leading to long-term dysfunction.
8. **Nerve Damage**: In rare cases, the dislocation can compress or damage nearby nerves, potentially leading to numbness, tingling, or weakness in the arm or hand.

Treatment typically involves either conservative management (rest, ice, physical therapy) or surgery, depending on the severity of the dislocation. Early intervention can help minimize these complications.

1. Can an AC joint dislocation lead to arthritis later in life?

Yes, an AC (acromioclavicular) joint dislocation can increase the risk of developing arthritis later in life. The AC joint, which is where the collarbone (clavicle) meets the shoulder blade (scapula), is important for shoulder function. After an injury like a dislocation, the joint's cartilage, ligaments, and surrounding structures can be damaged. This can lead to:

1. **Post-traumatic arthritis**: The disruption of the joint surface from the dislocation can cause uneven wear over time, leading to arthritis. The body's healing process might result in abnormal joint mechanics or joint instability, increasing wear and tear.
2. **Instability**: If the ligaments around the joint are stretched or torn during the dislocation, the AC joint may become unstable, which could contribute to further deterioration of the joint over time.
3. **Altered biomechanics**: A dislocated AC joint may lead to changes in the way the shoulder moves and functions, placing additional stress on the joint and increasing the likelihood of arthritis as the years go by.

Proper treatment, which may involve rest, physical therapy, or sometimes surgery, can help reduce the risk of long-term complications, but the possibility of arthritis does remain a concern for many individuals who have suffered from an AC joint dislocation.

1. How do I manage pain after an AC joint dislocation?

Managing pain after an AC (acromioclavicular) joint dislocation involves several approaches to reduce inflammation, promote healing, and manage discomfort. Here are some key steps:

**1. Rest and Immobilization**

- **Rest** is essential to prevent further injury. Avoid using the injured arm for activities that involve lifting or movement of the shoulder.
- You may be given a **sling** or brace to immobilize your shoulder and allow the joint to heal.

**2. Cold Therapy (Ice)**

- Apply **ice** to the injured area for 20–30 minutes every 2–3 hours during the first 48 hours. This helps reduce swelling and numb the pain.
- Be sure to wrap the ice pack in a cloth or towel to avoid direct contact with your skin.

**3. Pain Medications**

- **NSAIDs** (nonsteroidal anti-inflammatory drugs), like ibuprofen or naproxen, can help reduce pain and swelling. Always follow your doctor’s advice on dosage.
- **Acetaminophen** (Tylenol) can be used if you're concerned about the gastrointestinal side effects of NSAIDs.

**4. Elevation**

- Keep your shoulder elevated when sitting or lying down to help reduce swelling, though this can be difficult with a shoulder injury. A propped-up position on pillows can help.

**5. Gentle Range-of-Motion Exercises**

- After the initial healing period (typically 1–2 weeks), your healthcare provider may recommend **gentle stretching** and **range-of-motion exercises** to prevent stiffness.
- Avoid heavy lifting and forceful movements until your shoulder is fully healed.

**6. Physical Therapy**

- Once the acute pain subsides, **physical therapy** may be recommended to strengthen the muscles around the joint and improve flexibility. This will help prevent future injuries and restore full function to your shoulder.

**7. Heat Therapy (After Acute Stage)**

- After a few days to a week (once the initial swelling has decreased), **heat therapy** (such as a warm towel or heating pad) may be helpful in relaxing tight muscles and promoting blood circulation.

**8. Follow-up with Your Doctor**

- Regular follow-up visits with your doctor are important to assess healing progress. They may recommend imaging (like X-rays) if needed.

**9. Surgical Intervention (if needed)**

- In severe cases, or if conservative treatments don’t provide relief, **surgery** might be required. This is usually for high-grade AC joint dislocations.

If you feel any unusual symptoms like increased pain, numbness, or swelling that doesn’t improve, it’s important to consult your healthcare provider promptly. They can offer a tailored treatment plan and make sure you’re healing well.

Do you have any specific concerns or symptoms you’re dealing with after the injury?

1. When can I drive with an AC joint dislocation?

Driving with an AC (acromioclavicular) joint dislocation depends on several factors, including the severity of the injury and your comfort level. Here are some things to consider:

1. **Severity of the injury**:
   - If the dislocation is mild (a Grade 1 or Grade 2 sprain), you might be able to drive once you have enough range of motion and strength in your shoulder.
   - If it's a more severe dislocation (Grade 3 or higher), recovery may take longer, and you may need to avoid driving until the shoulder is sufficiently healed.
2. **Pain and Mobility**:
   - You’ll want to be able to move your arm, especially for steering, shifting, or using the brake and gas pedals comfortably and without pain.
   - If the pain is controlled with medication, ensure you're not drowsy or impaired.
3. **Consulting a Doctor**:
   - It's essential to consult with your healthcare provider. They will assess your healing progress and guide you based on how well you are recovering.
4. **Safety Considerations**:
   - Make sure you can perform all necessary movements to drive safely (turning the wheel, using signals, etc.).
   - If you're in pain or restricted in movement, it’s safer to avoid driving.

In most cases, people can resume driving once they regain enough mobility and strength in their shoulder, but it’s always best to check with your doctor for personalized advice.

1. Can I sleep on the injured side with an AC joint dislocation?

With an AC joint dislocation, it's generally not advisable to sleep on the injured side, especially in the early stages of recovery. Sleeping on the injured side can put additional pressure on the joint, causing pain and potentially delaying the healing process. It can also irritate the ligaments and soft tissues around the injury.

Instead, try sleeping on your opposite side or your back, using pillows to support your arm in a comfortable position. Some people find it helpful to use a pillow to prop up the injured arm to reduce strain on the joint.

Always follow your healthcare provider's advice, as the severity of your injury and your overall health may influence how best to manage your sleeping position.

1. How can I prevent another AC joint dislocation in the future?

Preventing another AC joint dislocation focuses on strengthening and protecting the shoulder, improving mobility, and being mindful of activities that might put stress on that joint. Here are some strategies to help prevent future dislocations:

**1. Strengthen the Surrounding Muscles:**

Strengthening the muscles around the shoulder can help stabilize the AC joint. Focus on:

- **Rotator cuff muscles**: These provide critical stability to the shoulder.
- **Deltoids**: These help protect the shoulder joint during motion.
- **Scapular stabilizers (e.g., rhomboids, traps, serratus anterior)**: A strong upper back helps stabilize the shoulder girdle.

**Exercises to consider:**

- **External rotations** (with resistance bands or weights)
- **Internal rotations**
- **Scapular retractions** (e.g., rows)
- **Overhead press** (moderate weight)

**2. Improve Range of Motion:**

A lack of flexibility or mobility can increase the risk of injury. Incorporating **dynamic stretches** before activity and **static stretches** after can maintain shoulder mobility and reduce tension on the AC joint.

**3. Protective Gear:**

In certain sports or activities, wearing shoulder protection (like a shoulder brace or harness) might help absorb shock or prevent direct trauma to the area.

**4. Avoid High-Risk Activities:**

Be cautious in activities where there’s a risk of falling or direct impact to the shoulder, such as contact sports (e.g., football, rugby, wrestling), extreme sports, or heavy lifting. Consider adjusting technique or avoiding particularly risky situations until you’re confident in your shoulder’s stability.

**5. Focus on Proper Technique:**

When engaging in activities like lifting weights, ensure your form is correct to avoid putting unnecessary stress on the shoulder. For example, when performing overhead presses, avoid overextending or improper posture that could destabilize the joint.

**6. Gradual Return to Activity:**

After a dislocation, allow enough time for full rehabilitation and recovery. Rushing back into strenuous activities can set you up for re-injury. A physical therapist can guide you through exercises to regain strength and motion progressively.

**7. Maintain Overall Body Conditioning:**

General fitness, including core stability, balance, and flexibility, contributes to shoulder health. It helps your body handle stresses and impacts more effectively.

**8. Consider Surgery for Severe Cases:**

If dislocations are frequent or very severe, surgery may be necessary to stabilize the AC joint. Surgical options like a **coracoclavicular ligament reconstruction** can prevent recurrence, but these are typically considered if non-surgical methods fail.

If you’re recovering from a previous dislocation, it’s essential to work closely with a physical therapist to address the specific issues that led to the injury and tailor a prevention plan for your situation. Would you like to explore any specific exercises or rehabilitation strategies further?

1. Will I regain full range of motion after an AC joint dislocation?

Regaining full range of motion after an AC (acromioclavicular) joint dislocation depends on several factors, including the severity of the dislocation, the treatment method, and how well you follow your rehabilitation plan.

For a **mild (Type I or II)** AC joint dislocation, conservative treatment (rest, ice, physical therapy, and possibly a sling) is often effective, and many people regain near or full range of motion with proper rehabilitation. This can take a few weeks to a few months.

For a **severe (Type III or higher)** dislocation, where there is more significant damage to the ligaments or the bones, recovery can be more complicated. Some people may still experience discomfort, reduced motion, or instability in the joint, even after surgery or extensive therapy.

Physical therapy plays a key role in the recovery process, helping to rebuild strength and improve flexibility. In some cases, however, if the joint doesn't fully heal or if there is ongoing pain, full range of motion may be harder to achieve.

In general:

- **Mild dislocations** often have good outcomes with full recovery.
- **Severe dislocations** may have a slower or more incomplete recovery, especially if not managed correctly.

If you're undergoing rehab or surgery, it's important to follow the advice of your healthcare provider to ensure the best possible recovery. Do you have any details about the severity of your dislocation or your current treatment plan?

1. What lifestyle modifications should I consider during my recovery from an AC joint dislocation?

Recovering from an AC (acromioclavicular) joint dislocation requires careful management to ensure healing and to prevent complications. Here are some lifestyle modifications you may want to consider during your recovery:

**1. Rest and Activity Modification:**

- **Avoid strenuous activities:** Activities that strain your shoulder or involve heavy lifting should be avoided until your doctor clears you. This includes weightlifting, sports like tennis or swimming, and any high-impact exercises.
- **Gentle Range of Motion Exercises:** Once your doctor or physical therapist approves, gentle range-of-motion exercises will help you regain mobility in your shoulder.
- **Gradual Return to Exercise:** As you recover, ease back into exercise with lower-impact activities. Walking or stationary biking might be good options early on.

**2. Pain and Swelling Management:**

- **Ice Therapy:** Apply ice packs to the affected shoulder to reduce swelling and pain, especially in the first few days after the injury.
- **Pain Relievers:** Over-the-counter pain relievers like ibuprofen or acetaminophen can help control pain and inflammation, but make sure to follow your doctor’s advice on dosage.

**3. Posture and Ergonomics:**

- **Good Posture:** Maintain proper posture to reduce strain on your shoulder joint. Avoid slouching, especially when sitting or standing for long periods.
- **Supportive Seating and Sleeping Positions:** Ensure your chair or desk promotes good posture. When sleeping, use pillows to support your arm in a comfortable position that avoids strain on the shoulder.

**4. Physical Therapy and Rehabilitation:**

- **Physical Therapy:** Your doctor may recommend physical therapy, which will focus on strengthening muscles around the shoulder joint, improving flexibility, and preventing re-injury.
- **Shoulder Strengthening Exercises:** Once healing allows, you'll want to engage in exercises that strengthen the rotator cuff and upper back muscles to support the shoulder joint and avoid future injuries.

**5. Nutrition and Hydration:**

- **Adequate Protein and Calcium:** To help the healing process, make sure you are consuming enough protein, calcium, and Vitamin D to support tissue repair and bone health.
- **Stay Hydrated:** Proper hydration can help reduce muscle stiffness and promote healing.

**6. Post-Surgery Modifications (If applicable):**

- If surgery was required, you may need to follow more strict activity restrictions. Your arm may be in a sling or brace for several weeks, and follow-up appointments will help ensure proper healing.

**7. Psychological and Emotional Support:**

- **Mental Well-being:** It can be frustrating to be limited by an injury, so take care of your mental health as well. You may benefit from relaxation techniques or simply staying engaged in hobbies that don't require heavy shoulder use.

Always follow your healthcare provider’s recommendations and avoid rushing the healing process. This injury can take some time to fully recover from, so patience is key to ensuring a complete and safe return to activity.
